# Traumatic Cataract Following Accidental HIFU Exposure of the Upper Eyelid: A Rare Ocular Complication—Case Report

**DOI:** 10.3390/diagnostics16213559

**Published:** 2026-10-29

**Authors:** Aleksandra Górska, Dorota Wyględowska-Promieńska, Wojciech Luboń, Adrian Smędowski, Sebastian Sirek, Mikołaj Guzikowski, Mariola Dorecka

**Affiliations:** 1 Department of Ophthalmology, Prof. K. Gibiński University Clinical Center, Medical University of Silesia in Katowice, 40-055 Katowice, Poland; 2 Department of Ophthalmology, Faculty of Medical Sciences in Katowice, Medical University of Silesia in Katowice, 40-055 Katowice, Poland; 3 Students’ Scientific Society, Department of Ophthalmology, Faculty of Medical Sciences in Katowice, Medical University of Silesia, 40-055 Katowice, Poland

**Keywords:** HIFU, traumatic cataract, ocular complications, cosmetic ultrasound, eyelid, anterior segment OCT, ocular imaging

## Abstract

High-intensity focused ultrasound (HIFU) is increasingly used for noninvasive facial tightening, but inadvertent exposure of the periocular region may injure ocular structures. We present a 34-year-old woman who developed sudden visual deterioration approximately 30 min after facial HIFU when the applicator accidentally moved onto the right upper eyelid. Best-corrected visual acuity (BCVA), measured using Snellen charts, was 0.2 in decimal scale in the right eye and 1.0 in the left eye, with intraocular pressure of 17 and 14 mmHg, respectively. Slit-lamp examination revealed a slightly dilated, sluggish pupil and dense inferior lens opacity with a rosette-shaped opacity involving the posterior lens, while the eyelid skin and cornea remained intact. Anterior segment optical coherence tomography (AS-OCT) and ultrasound biomicroscopy showed no capsular rupture; ultrasound biomicroscopy additionally demonstrated separation of cortical lens material from the posterior capsule. BCVA progressively decreased to 0.04 before cataract surgery. Phacoemulsification with implantation of a non-diffractive spiral intraocular lens was performed two months after the HIFU exposure. On postoperative day 1, uncorrected visual acuity was 1.0 at distance and 0.5 at near, with intraocular pressure of 16 mmHg, and these findings remained stable during six months of follow-up. Posterior segment OCT and visual field testing showed no clinically relevant abnormalities. The images illustrate a characteristic pattern of traumatic cataract following inadvertent periocular HIFU exposure and the multimodal imaging used for diagnosis and follow-up. This case emphasizes that lens injury may occur despite an intact cornea and eyelid and supports strict avoidance of direct HIFU application to the eyelids, together with appropriate ocular protection.

**Figure 1:**
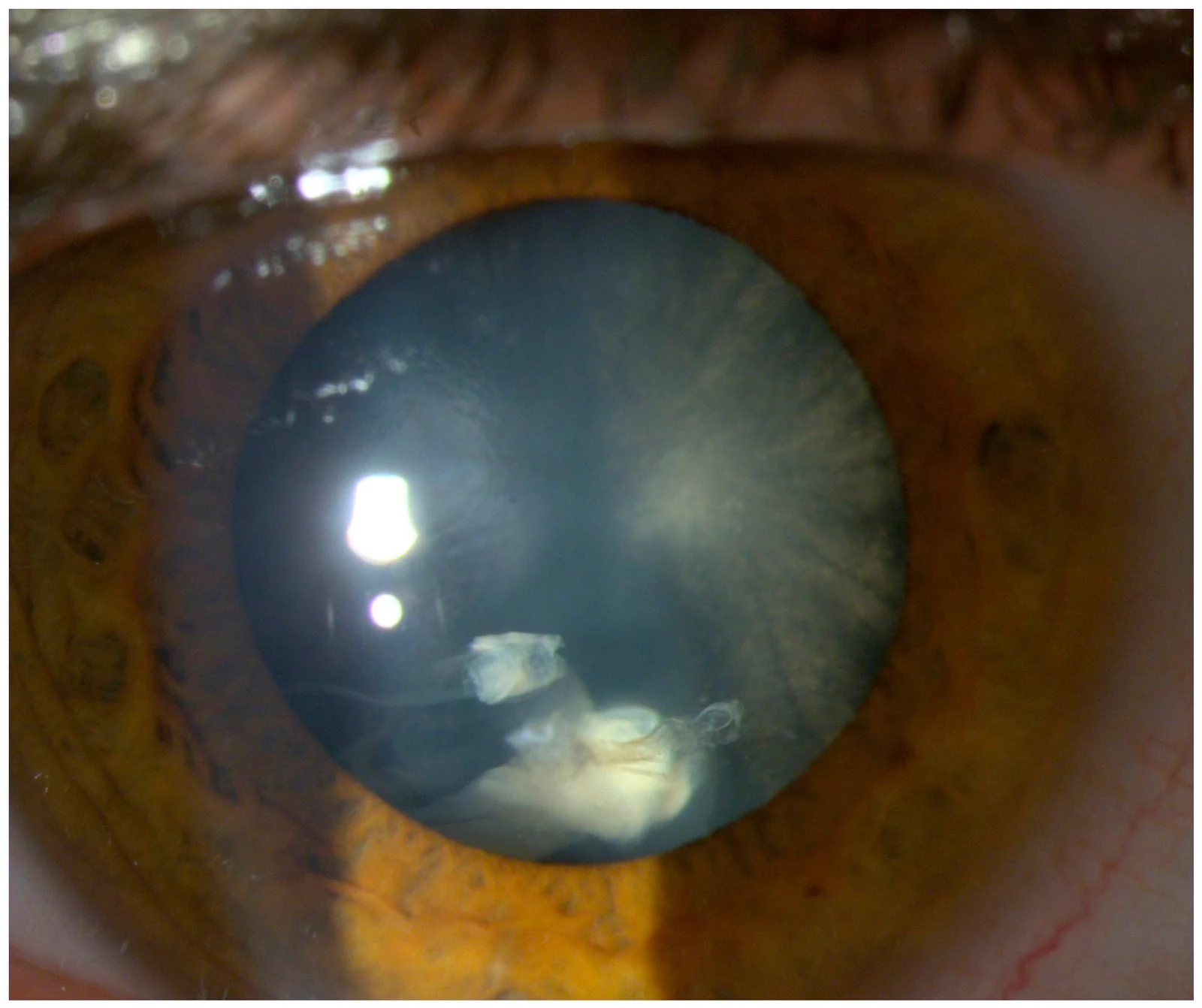
Preoperative slit-lamp photograph of the right eye in a 34-year-old woman obtained after sudden visual deterioration approximately 30 min following a facial HIFU procedure. During treatment, the applicator accidentally shifted onto the right upper eyelid. A dense, sectoral, whitish opacity is visible in the inferior portion of the lens, with additional posterior lens opacity. The eyelid skin and corneal surface remained clinically intact. At presentation, BCVA was 0.2 in the right eye and 1.0 in the left eye, and intraocular pressure was 17 and 14 mmHg, respectively. The lens opacity progressed and reached its final appearance within 2 days of exposure, after which it remained stable. BCVA subsequently declined to 0.04 in the affected eye before cataract surgery. The close temporal relationship to HIFU exposure, together with the absence of previous ocular disease, ocular surgery, trauma, or active infection, supported the diagnosis of HIFU-associated traumatic cataract. HIFU produces a focal thermal effect in the target tissue; inadvertent periocular exposure may therefore affect deeper ocular structures despite preservation of the superficial tissues [1,2,3,4,5].

**Figure 2:**
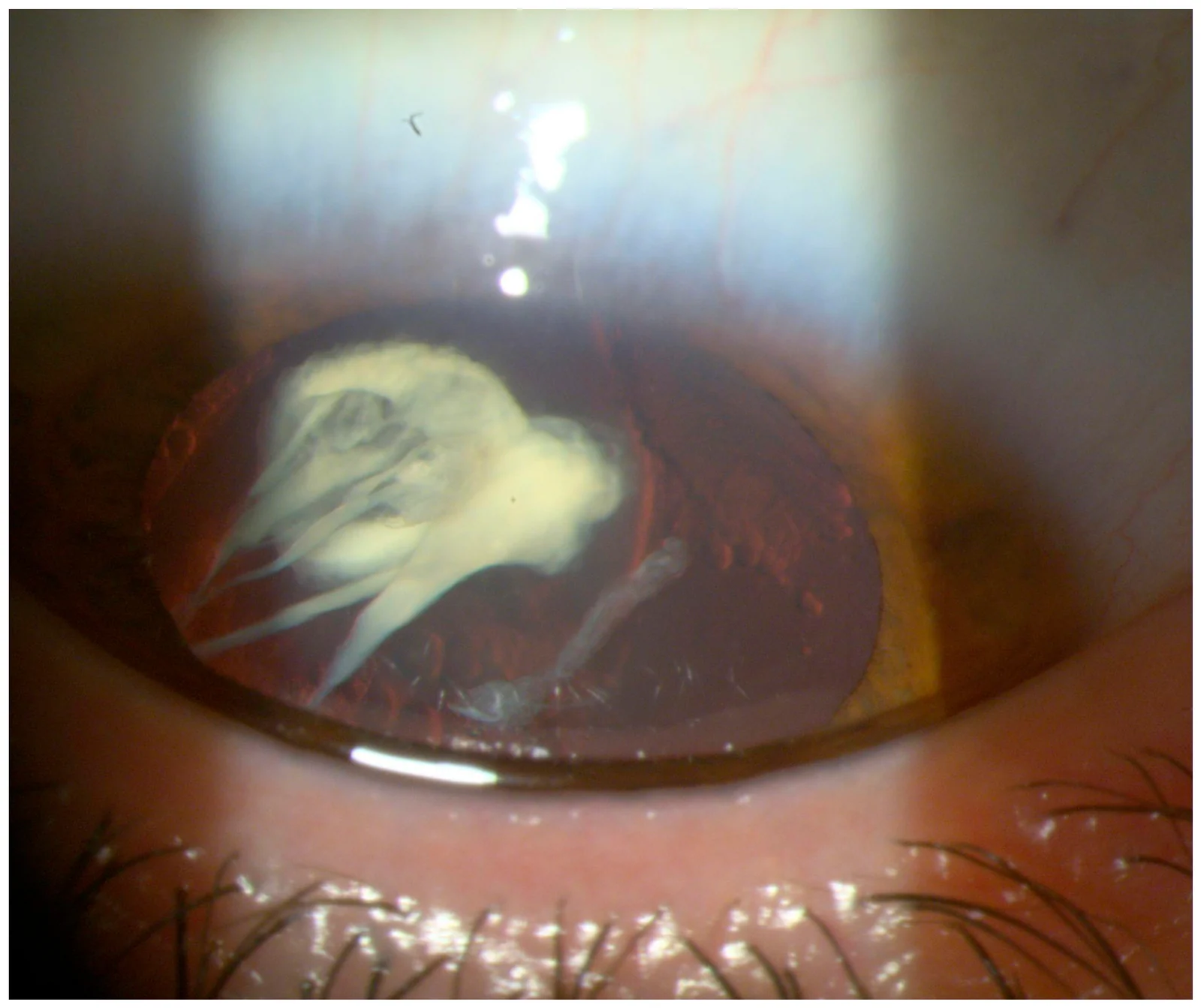
Retroillumination of the right eye demonstrating the characteristic morphology of the lens opacity. A dense inferior cataract is accompanied by a posterior, rosette-shaped opacity.

The abrupt onset after accidental eyelid exposure supports an association with HIFU-related lens injury. Lens opacities following cosmetic HIFU procedures have been described previously [5,6,7,8].

The patient had no history of previous ocular surgery or trauma. The absence of corneal injury and the characteristic lens morphology are notable because the energy was applied externally to the upper eyelid rather than directly to the globe. The technical parameters of the HIFU device used in this case—including manufacturer and model, frequency, focal depth, power, and delivered energy—were unavailable, limiting any attempt to relate the observed injury to a specific exposure threshold.

**Figure 3:**
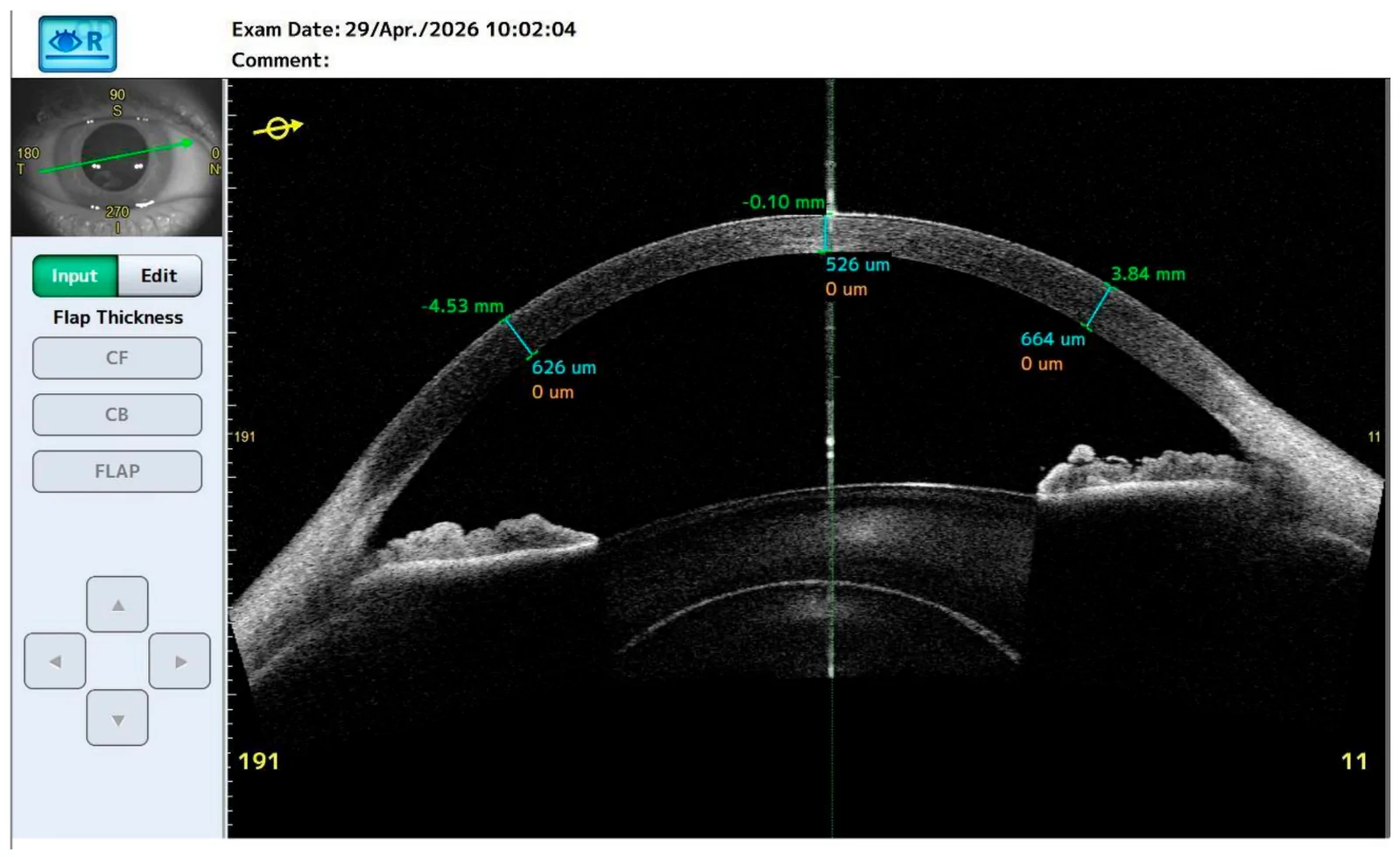
Preoperative anterior segment optical coherence tomography (AS − OCT; CASIA2, Tomey Corporation, Nagoya, Japan) of the right eye, showing preserved corneal contour and the anterior segment configuration in the presence of lens opacity.

**Figure 4:**
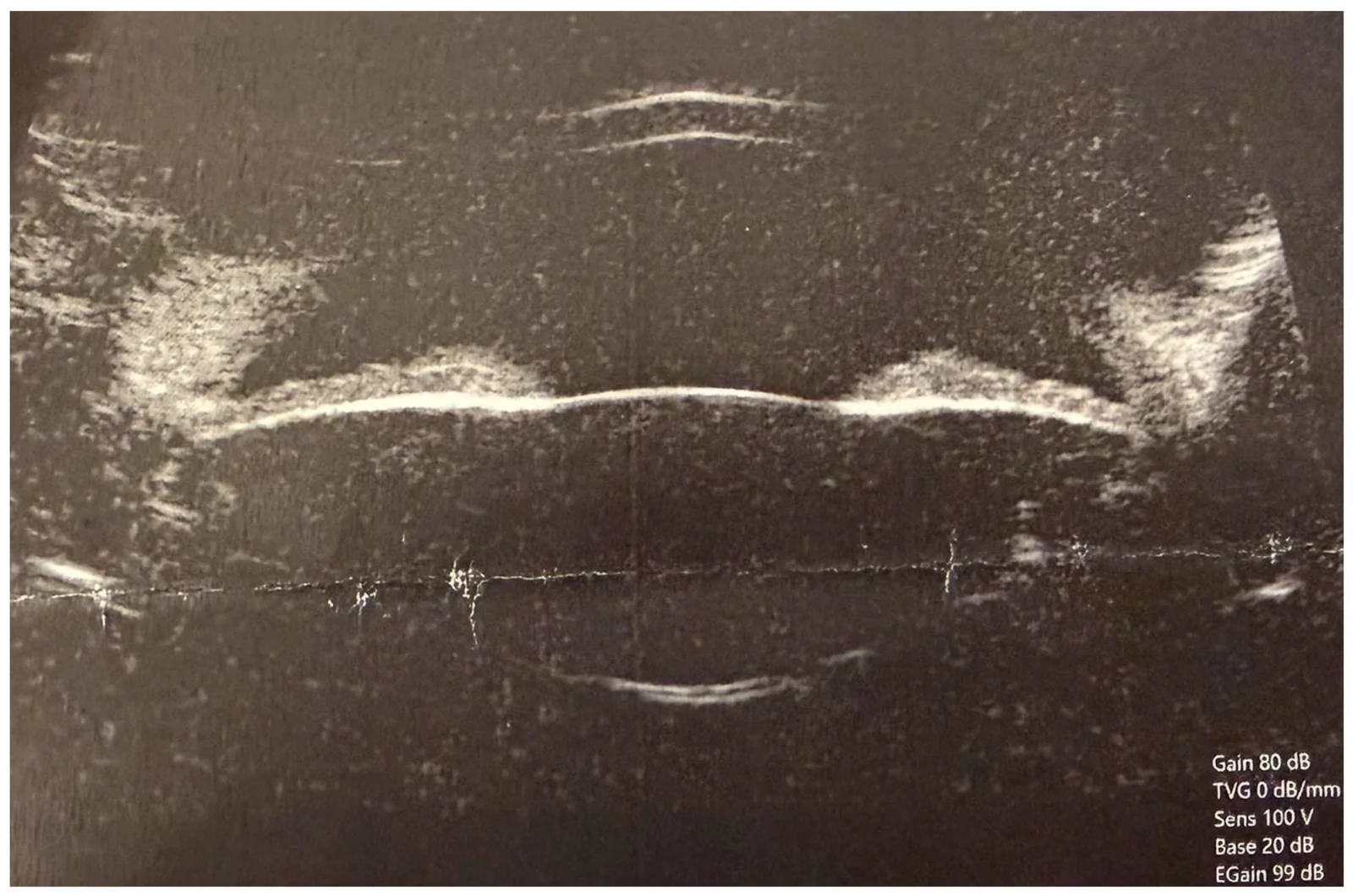
Ultrasound biomicroscopy (UBM, Sonomed Escalon, New Hyde Park, NY, USA) showed separation of cortical lens material from the posterior capsule, with no evidence of capsular rupture. These findings suggest that significant lens injury can occur without demonstrable gross capsular disruption or visible damage to superficial ocular structures. These examinations complemented the slit-lamp assessment and informed preoperative evaluation. Multimodal imaging may help assess intraocular injury after periocular HIFU exposure despite an apparently intact ocular surface [3,4]. Nevertheless, the exact mechanism remains uncertain, particularly because the technical parameters of the HIFU device were unavailable.

**Figure 5:**
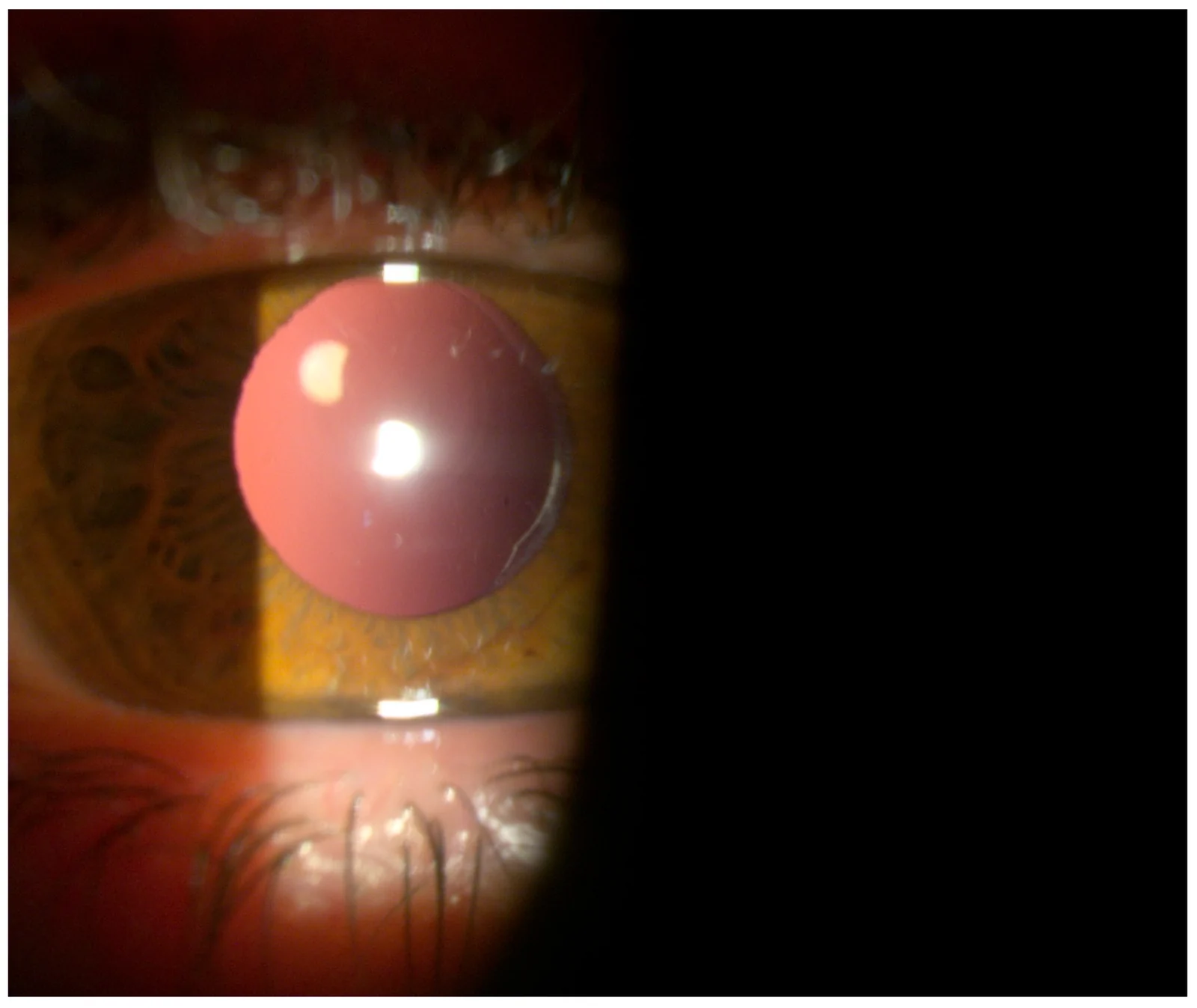
Slit-lamp photograph of the right eye 7 days after phacoemulsification and implantation of a non-diffractive spiral intraocular lens (RayOne Galaxy; Rayner, Worthing, UK). The previously opaque lens has been removed and a clear intraocular lens is centered within the capsular bag, with a well-defined red reflex and no visible postoperative corneal complication. Cataract surgery was performed two months after the HIFU exposure. On postoperative day 1, uncorrected visual acuity was 1.0 at distance and 0.5 at near, with intraocular pressure of 16 mmHg. Visual acuity remained stable during six months of follow-up.

**Figure 6:**
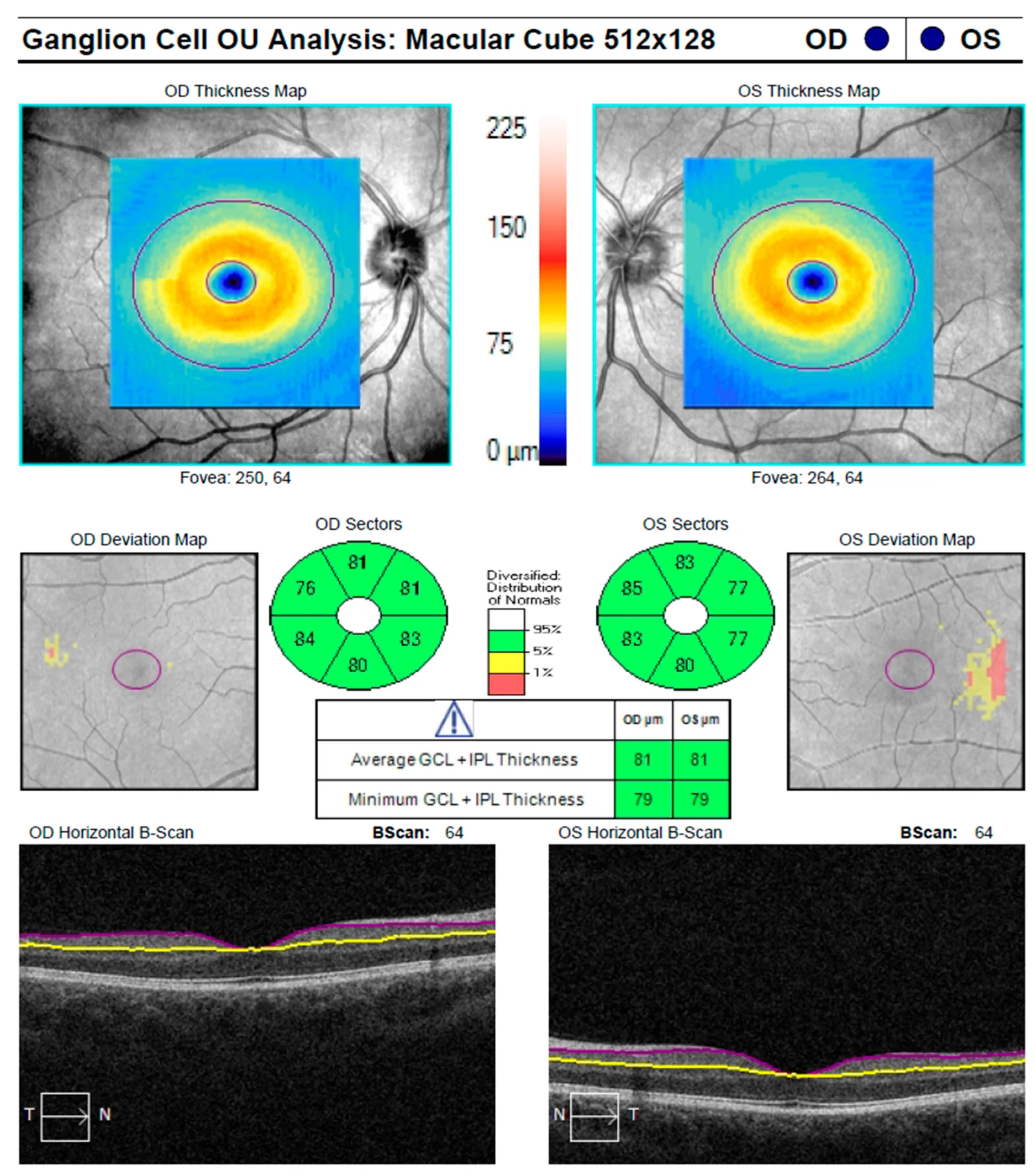
Posterior segment optical coherence tomography (Cirrus HD-OCT, ZEISS, Germany) obtained during follow-up.

The displayed ganglion cell analysis includes both eyes and shows preserved foveal contour, with sectoral and average ganglion cell–inner plexiform layer thicknesses within the device’s normative range.

At six months after cataract surgery, the patient maintained uncorrected visual acuity of 1.0 at distance and 0.5 at near, assessed using Jaeger charts on a decimal scale, in the right eye, with an intraocular pressure of 15 mmHg.

These findings, together with visual recovery after cataract surgery, support the lens as the principal site of clinically apparent injury.

**Figure 7:**
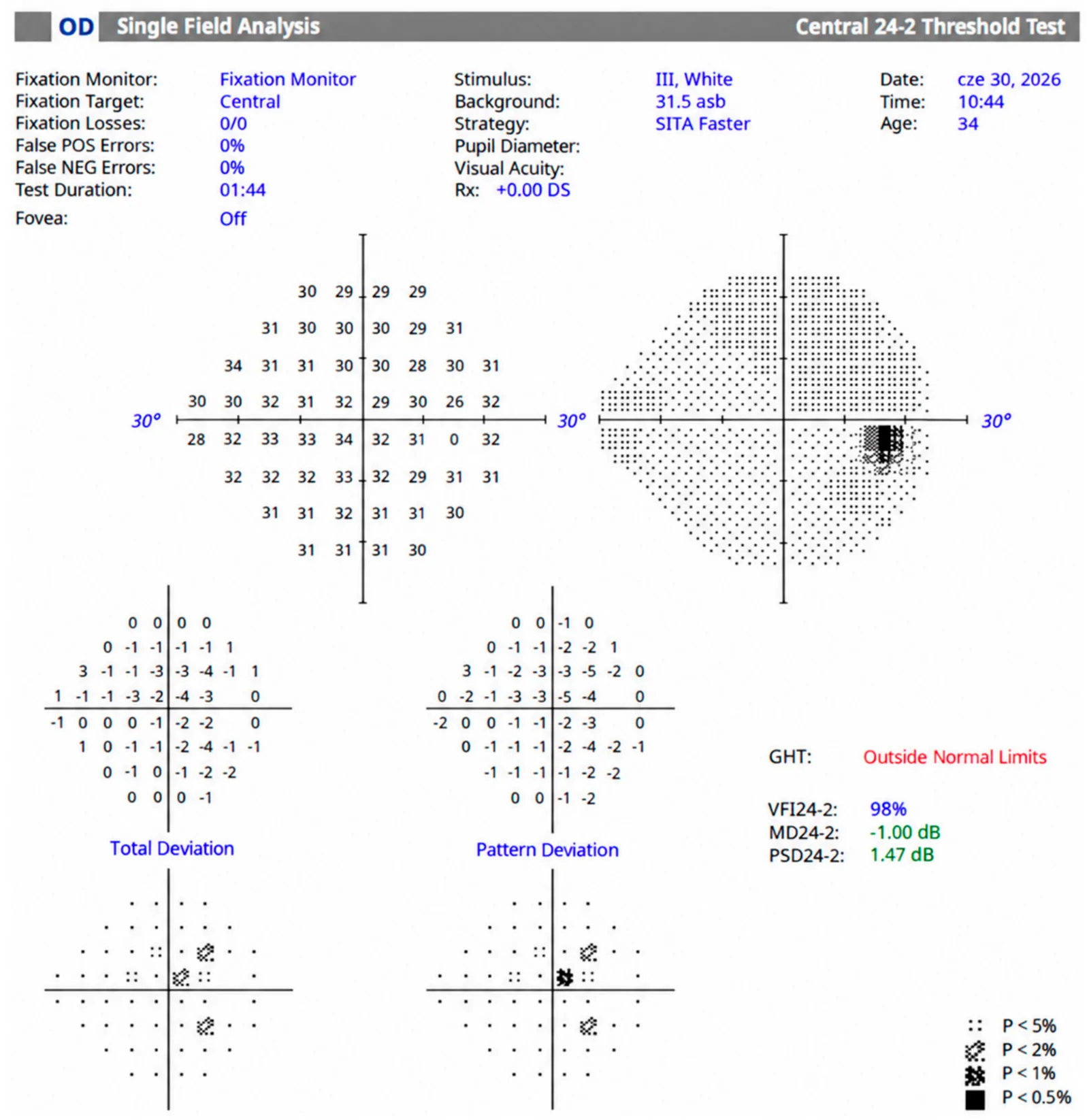
Automated visual field examination (Humphrey Field Analyzer; ZEISS, 24−2 SITA Faster strategy) of the right eye during follow-up. The examination was performed using the 24 − 2 SITA Faster strategy, with a false-positive response rate of 0%. The reported parameters were a visual field index (VFI) of 98%, mean deviation (MD) of −1.00 dB, and pattern standard deviation (PSD) of 1.47 dB.

The near-normal global perimetric indices were consistent with largely preserved overall visual field sensitivity at follow-up. The complete imaging sequence—from preoperative slit-lamp and retroillumination photographs through AS-OCT and postoperative structural and functional assessment—illustrates the diagnostic value of multimodal ocular imaging in this rare complication.

## Data Availability

No new datasets were created or analyzed in this study. The data supporting the findings of this case report are contained within the article. Additional clinical information and images are not publicly available due to patient privacy and ethical restrictions.
